# STAT3 Is Activated by JAK2 Independent of Key Oncogenic Driver Mutations in Non-Small Cell Lung Carcinoma

**DOI:** 10.1371/journal.pone.0030820

**Published:** 2012-02-02

**Authors:** Brendan D. Looyenga, Danielle Hutchings, Irene Cherni, Chris Kingsley, Glen J. Weiss, Jeffrey P. MacKeigan

**Affiliations:** 1 Systems Biology, Van Andel Research Institute, Grand Rapids, Michigan, United States of America; 2 Lung Cancer Unit, Cancer & Cell Biology Division, The Translational Genomics Research Institute (TGen), Phoenix, Arizona, United States of America; 3 Diabetes, Cardiovascular & Metabolic Diseases Division, TGen, Phoenix, Arizona, United States of America; 4 Virginia G. Piper Cancer Center Clinical Trials, Scottsdale Healthcare, Scottsdale, Arizona, United States of America; University of Texas-M.D. Anderson Cancer Center, United States of America

## Abstract

Constitutive activation of STAT3 is a common feature in many solid tumors including non-small cell lung carcinoma (NSCLC). While activation of STAT3 is commonly achieved by somatic mutations to JAK2 in hematologic malignancies, similar mutations are not often found in solid tumors. Previous work has instead suggested that STAT3 activation in solid tumors is more commonly induced by hyperactive growth factor receptors or autocrine cytokine signaling. The interplay between STAT3 activation and other well-characterized oncogenic “driver” mutations in NSCLC has not been fully characterized, though constitutive STAT3 activation has been proposed to play an important role in resistance to various small-molecule therapies that target these oncogenes. In this study we demonstrate that STAT3 is constitutively activated in human NSCLC samples and in a variety of NSCLC lines independent of activating KRAS or tyrosine kinase mutations. We further show that genetic or pharmacologic inhibition of the gp130/JAK2 signaling pathway disrupts activation of STAT3. Interestingly, treatment of NSCLC cells with the JAK1/2 inhibitor ruxolitinib has no effect on cell proliferation and viability in two-dimensional culture, but inhibits growth in soft agar and xenograft assays. These data demonstrate that JAK2/STAT3 signaling operates independent of known driver mutations in NSCLC and plays critical roles in tumor cell behavior that may not be effectively inhibited by drugs that selectively target these driver mutations.

## Introduction

Non-small cell lung carcinoma (NSCLC) is characterized by a remarkable variety of genomic alterations and point mutations that collectively disrupt the normal molecular programs which regulate growth and survival of the lung epithelium [1], [2]. Despite the genomic complexity of individual lung tumors, however, it appears that most oncogenic programs are driven by only a few key mutations, which are required to maintain the viability and proliferation of tumor cells. The key “driver oncogenes” that have been associated with NSCLC include Kirsten rat sarcoma viral oncogene homolog (KRAS) and several receptor tyrosine kinases (RTK) such as the epidermal growth factor receptor (EGFR), platelet-derived growth factor receptor (PDGFR), anaplastic lymphoma kinase (ALK) and hepatocyte growth factor receptor (HGFR/MET) [3]. All of these oncogenes converge upon a number of signaling pathways that have been recurrently implicated across the spectrum of solid tumors.

While effective treatments for KRAS-driven tumors have generally been lacking, significant progress has been made in the development of tyrosine kinase inhibitors (TKI) that selectively block the activity of constitutively activated RTKs [4]. Perhaps the best example of this therapeutic strategy is the development of small-molecule inhibitors of EGFR, including gefitinib and erlotinib [5], [6]. Patients with specific activating mutations to EGFR respond favorably to these drugs, though most eventually relapse or progress due to a variety of acquired resistance mechanisms [7]. Recent work in several different cancer types has indicated a particular role for the microenvironment—including both stromal and inflammatory cells—in acquired drug resistance, though the specific signaling mechanisms associated with this process remain incompletely understood [4], [8], [9].

Of the many different microenvironment signaling cues that contribute to tumor cell survival, those that induce the activation of signal transducer and activator of transcription (STAT) family proteins appear to be particularly important in tumor drug resistance [10], [11]. STAT3, which can be activated by a variety of growth factor and inflammatory signals from the microenvironment, has been implicated in both primary and acquired resistance to chemotherapy [12], [13], [14]. In addition, STAT3 was recently identified as a target in a large-scale synthetic lethal screen designed to isolate compounds that synergize with EGFR inhibitors [15]. Several previous studies have demonstrated that STAT3 is required for efficient cellular transformation by an array of well-characterized oncogenes including Ras, v-Src, SV40 T-antigen, and EGFR, further validating the importance of STAT3 in cancer biology [16], [17], [18], [19], [20].

STAT3 is primarily activated by tyrosine (Y705) phosphorylation, which can be mediated by a number of tyrosine kinases included those of the SRC and JAK families [10], [21]. Tyrosine-phosphorylated STAT3 molecules form homodimers that traffic to the nucleus, bind DNA and activate transcription of key target genes involved in cellular proliferation, survival and migration. While other phosphorylation-independent functions have been described for STAT3, it is unclear whether they play an important role in the pro-oncogenic function of STAT3 in human tumors [22]. Conversely, many studies have demonstrated the importance of tyrosine-phosphorylation in the oncogenicity of STAT3, suggesting that this modification is critical for its role in cellular transformation [10], [21]. Notably, tyrosine-phosphorylated STAT3 has been found in up to 50% of late-stage NSCLC, suggesting a possible role in the etiology or progression of this cancer [10], [23], [24].

In this study we sought to identify signaling pathways that remain active after treatment of NSCLC lines with TKIs. Our data demonstrate that STAT3 activity is unaffected by TKI treatment in several different cellular contexts. Furthermore, we show that STAT3 phosphorylation at Y705 is primarily regulated by the gp130/JAK2 signaling complex, and is constitutively active in several different NSCLC cell lines as well as in 22% of early-stage NSCLC samples. Treatment of NSCLC lines with the JAK2 inhibitor ruxolitinib inhibits anchorage independent growth and slows xenograft tumor formation, suggesting that selected patients with NSCLC may benefit from treatment with JAK2 inhibitors in addition to TKI.

## Results

### Targeted Kinase Inhibitors Fail to Downregulate STAT3 Phosphorylation

**Figure 1:**
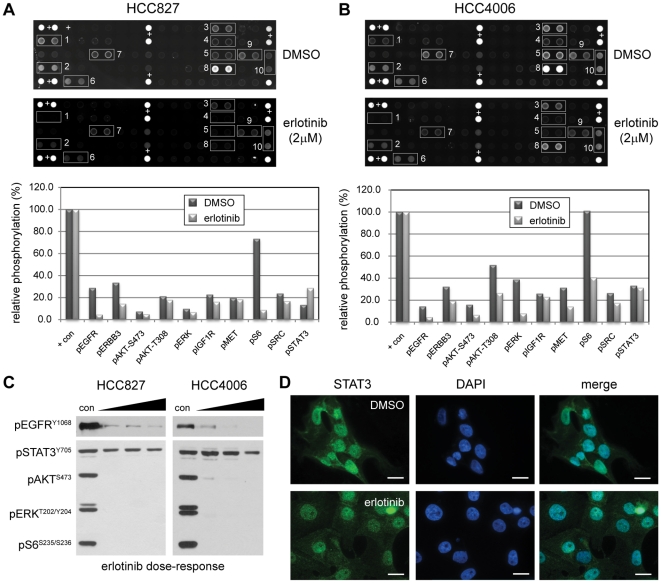
Erlotinib fails to inhibit STAT3 activation in EGFR mutant NSCLC cell lines. **A–B**, Protein lysates from HCC827 (A) and HCC4006 (B) cells treated with DMSO (vehicle) or 2 µM erlotinib for 8 hours were analyzed with antibody microarrays to detect phosphorylation status of receptor tyrosine kinases and key cell signaling proteins. Fluorescent signals for the indicated spots were background subtracted and normalized to positive control spots (indicated with +) on the array. Data is shown as the percentage of average fluorescence for each set of duplicate spots relative to the average fluorescence of ten positive control spots for each array. Data on each graph is ordered according to the number (1–10) indicated on each array. **C**, HCC827 and HCC4006 cells were treated for one hour with DMSO (con) or a 2-fold escalating dose of erlotinib ranging from 0.25 to 1.0 µM (left-right). The phosphorylation status of EGFR (pEGFR^Y1068^), STAT3 (pSTAT3^Y705^), AKT (pAKT^S473^), ERK (pERK^T202/Y204^), and S6 (pS6^S235/S236^) was evaluated by immunoblot. Equal amounts of protein (20 µg) were added for each sample. **D**, HCC4006 cells were treated for two hours with DMSO or 1.0 µM erlotinib, then fixed and stained with antibodies for total STAT3. Cells were counter-stained with DAPI to indicate nuclear accumulation of STAT3. Scale bars, 20 µm.

In order to identify signaling pathways that fail to respond to targeted tyrosine kinase inhibitors (TKIs), we treated EGFR-mutant cell lines (HCC827 and HCC4006) with the small molecule inhibitor erlotinib and assayed multiple signaling pathways using a phosphoprotein array (Fig. 1A–B). As expected, both EGFR and several of its downstream effectors—including PI-3-kinase (PI3K), mTOR and ERK—are deactivated by erlotinib treatment. However, we also observed that STAT3 remains activated in both cell lines after erlotinib treatment (Fig. 1A–B, array location 10). Immunoblot analysis of these pathways confirmed the findings of the phosphoprotein array, suggesting that STAT3 is activated independent of EGFR in these NSCLC cells (Fig. 1C). Furthermore, inhibition of EGFR with erlotinib failed to change the nuclear localization of STAT3 in HCC4006 cells, indicating that STAT3 remains active and appropriately localized in the absence of signaling by EGFR (Fig. 1D)

**Figure 2:**
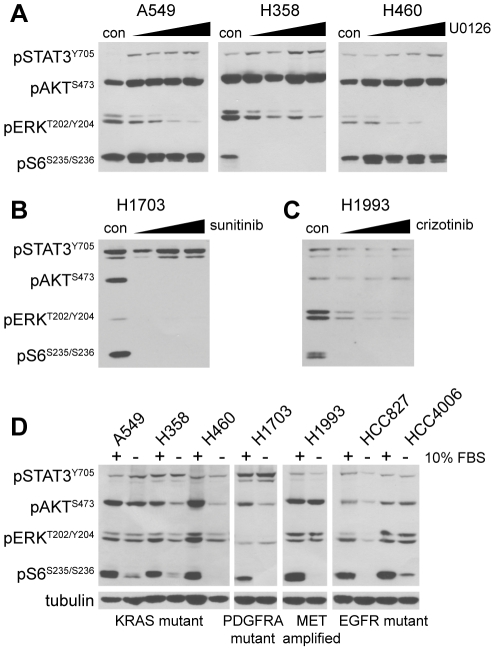
Targeted kinase inhibitors fail to downregulate STAT3 phosphorylation in NSCLC cell lines with diverse driver mutations. **A**, KRAS mutant NSCLC cell lines (A549, NCI-H358 and NCI-H460) were treated for one hour with DMSO (con) or a 2-fold escalating dose of the MEK1/2 inhibitor U0126 ranging from 0.625 to 5.0 µM (left-right). The phosphorylation status of STAT3 (pSTAT3^Y705^), AKT (pAKT^S473^), ERK (pERK^T202/Y204^), and S6 (pS6^S235/S236^) was evaluated by immunoblot. Equal amounts of protein (20 µg) were added for each sample. **B**, PDGFRA amplified/mutant NSCLC cells (NCI-H1703) were treated for one hour with DMSO (con) or a 2-fold escalating dose of the PDGFRA inhibitor sunitinib ranging from 0.625 to 2.5 µM (left-right). Phospho-protein status was evaluated in 20 µg of protein per sample as in A. **C**, MET amplified NSCLC cells (NCI-H1993) were treated for one hour with DMSO (con) or a 2-fold escalating dose of the MET/ALK inhibitor crizotinib ranging from 0.25 to 1.0 µM (left-right). Phospho-protein status was evaluated in 20 µg of protein per sample as in A. **D**, The indicated NSCLC lines were plated at fixed densities and allowed to adhere for 24 hours. Cells were then treated for 18 hours in normal media with (+) or without (−) 10% fetal bovine serum (FBS). Phospho-protein status was evaluated in 20 µg of protein per sample as in A. Even sample loading is indicated by replicate immunoblot for beta-tubulin.

To extend these findings, we analyzed activation of STAT3 in five other NSCLC lines with diverse “driver oncogenes” commonly found in non-squamous NSCLC (Table S1). Treatment of KRAS mutant cells (A549, NCI-H358, NCI-H460) with the MEK1/2 inhibitor U0126 readily inhibits activation of the MAPK pathway (ERK1/2), but fails to inhibit STAT3 activation (Fig. 2A). In fact, we observed that MEK1/2 inhibition actually increases STAT3 phosphorylation, suggesting that STAT3 may be activated in response to a decrease in MEK activity downstream of KRAS signaling (Fig. 2A) [25].

We also analyzed the activation of STAT3 in NSCLC lines bearing genomic amplification of the tyrosine kinases PDGFRA (NCI-H1703) and MET (NCI-H1993), both of which are found to be amplified or mutated in NSCLC [26], [27]. Inhibition of PDGFRA with the TKI sunitinib and MET with the TKI crizotinib potently blocks the activation of PI3K, RAS and mTOR pathways, as demonstrated by decreased phosphorylation of the downstream effector proteins AKT, ERK1/2 and S6 in both cell lines (Fig. 2B–C). Even at relatively high doses, however, neither sunitinib nor crizotinib significantly decreases the phosphorylation of STAT3 compared to these other RTK signaling effectors.

To demonstrate that STAT3 activation in each of the seven NSCLC lines occurs independent of serum stimulation, we plated cells at a fixed density, allowed them to adhere overnight and then treated them with or without fetal bovine serum (FBS, 10%) for 24 hours. Immunoblot analysis of signaling protein phosphorylation in these conditions indicates that activation of AKT, ERK1/2 and S6 is variably dependent upon serum stimulation, depending on cell type, while STAT3 activation occurs independent of serum stimulation (Fig. 2D). These data collectively suggest that STAT3 is activated independent of growth factor signaling pathways or activated RTKs in NSCLC, and is therefore insensitive to kinase inhibitors that target these pathways.

### STAT3 is Activated via Autocrine Signaling by IL-6 Family Ligands

**Figure 3:**
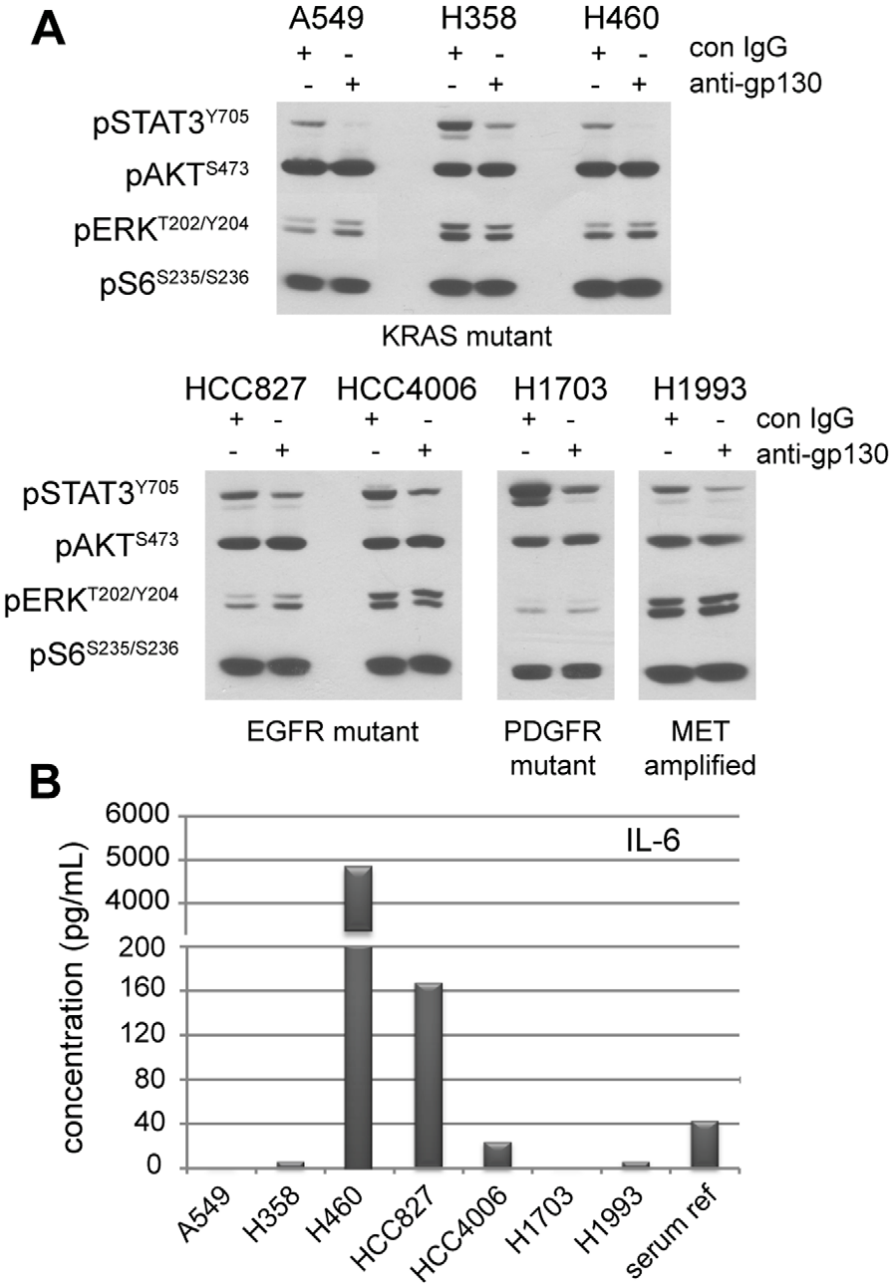
STAT3 phosphorylation is mediated by IL-6 family cytokines in NSCLC cell lines. **A**, The indicated NSCLC lines were plated at fixed densities and allowed to adhere overnight in serum-containing media. Cells were then treated for 24 hours in serum-free media containing anti-gp130 neutralizing antibody or a control mouse IgG (2.0 µg/mL each). Protein lysates were harvested and the phosphorylation status of STAT3 (pSTAT3^Y705^), AKT (pAKT^S473^), ERK (pERK^T202/Y204^), and S6 (pS6^S235/S236^) was evaluated by immunoblot. Equal amounts of protein (30 µg) were added for each sample. **B**, NSCLC lines were plated at fixed densities and allowed to adhere overnight in serum-containing media. Cells were then serum-starved, and conditioned media was collected at 48 hours. Secreted interleukin-6 (IL-6) levels were measured from the indicated cell lines using a bead-based immunoassay platform. Values shown are averages of duplicate measurements for each sample.

Previous studies have suggested that STAT3 is primarily activated in autocrine fashion by the interleukin-6 (IL-6) family of cytokines in NSCLC [14], [24]. These cytokines all bind and activate the common co-receptor gp130, which can be blocked using neutralizing antibodies that prohibit binding of all IL-6 superfamily cytokines to the extracellular region of the receptor. We found that gp130 neutralization effectively decreases STAT3 phosphorylation in all seven NSCLC lines regardless of their driver mutation status, suggesting that one or more IL-6 family ligands is responsible for autocrine activation of STAT3 in these cell lines (Fig. 3A).

The IL-6 family of cytokines contains seven different members, though only IL-6 itself has been previously implicated in NSCLC [14], [24]. To determine whether IL-6 was expressed in the seven NSCLC lines, we measured the production of IL-6 and several other inflammatory cytokines in conditioned media after 48 hours of culture in serum-free conditions (Table S2). Of the seven NSCLC lines tested, however, only three (NCI-H460, HCC827 and HCC4006) produce appreciable levels of IL-6 relative to normal human serum (Fig. 3B). These findings are consistent with mRNA expression data, which demonstrate that the four NSCLC lines lacking detectable production of IL-6 ligand (A549, NCI-H358, NCI-H1703 and NCI-H1993) also fail to express IL-6 mRNA (Fig S1A).

At this time it is unclear which other IL-6 family ligand is responsible for STAT3 activation in the four cell lines that lack detectlable IL-6 expression. Of the seven IL-6 family ligands present in the human genome, only cardiotrophin-like cytokine factor 1 (CLCF1) is expressed at levels similar to or above IL-6 in the NSCLC lines we tested by quantitative RT-PCR (Fig S1A). Because commercial tests for CLCF1 are not available, we developed a sandwich ELISA for CLCF1 that can quantify soluble ligand in serum-free tissue culture media (Fig S1B). Using this assay we were able to detect CLCF1 in the conditioned media of several of the NSCLC lines (Fig S1C). The significance of this finding is unclear, however, since lung epithelial cells lack the primary receptor for this ligand (ciliary neurotrophic factor receptor, CNTFR) and fail to respond to recombinant CLCF1 (Fig S1D). Further studies will be required to accurately measure secretion of other IL-6 family ligands and to determine which of them are capable of activating STAT3 via the gp130 receptor in NSCLC.

### STAT3 is Primarily Activated by JAK2 in NSCLC

**Figure 4:**
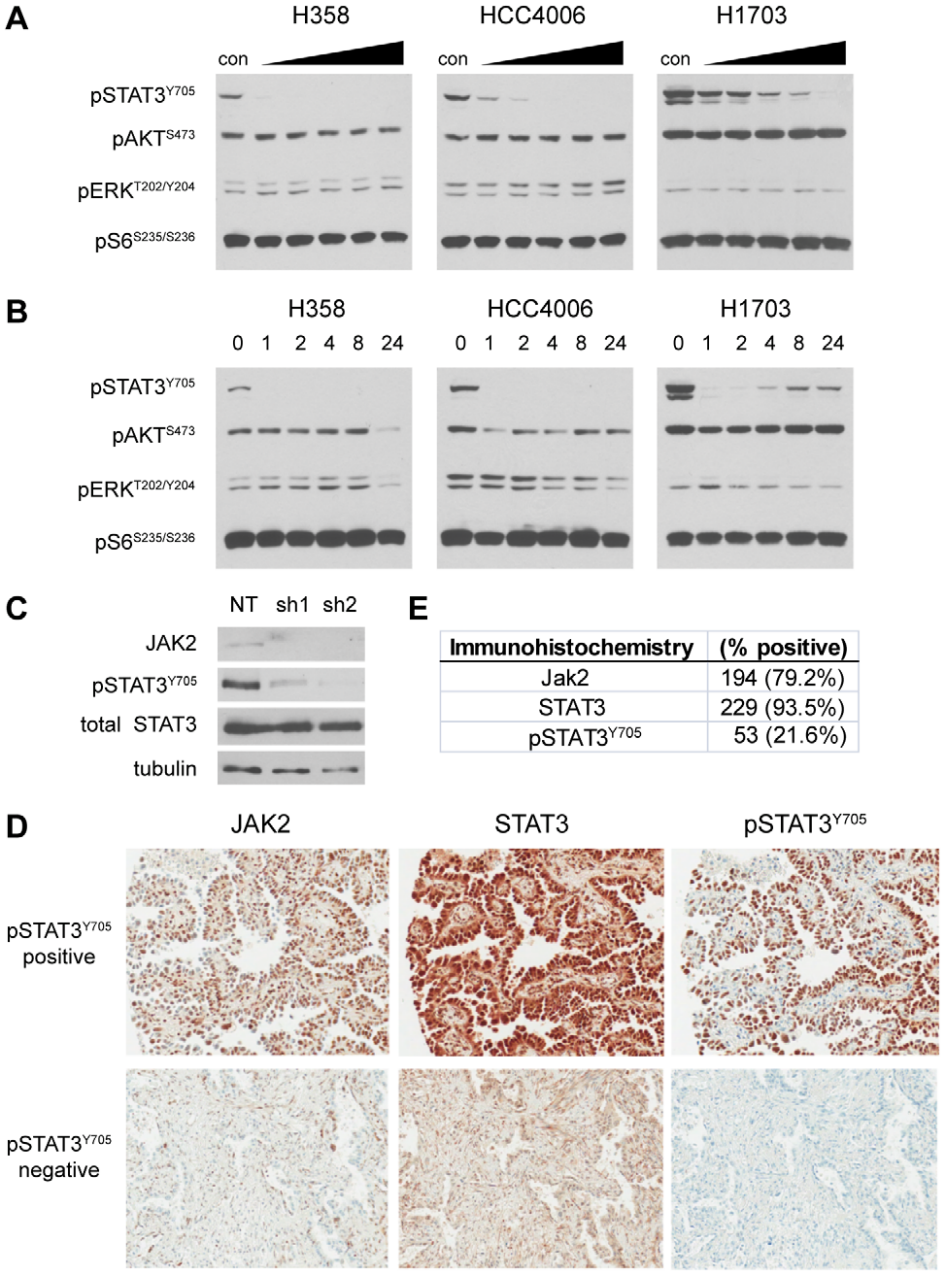
STAT3 phosphorylation is mediated by the tyrosine kinase JAK2 in NSCLC. **A**, The indicated NSCLC lines were plated at a fixed density and allowed to adhere overnight. Cells were then treated for 16 hours with DMSO (con) or a 2-fold escalating dose of the JAK1/2 inhibitor ruxolitinib ranging from 0.25 to 4.0 µM (left-right). Protein lysates were harvested and the phosphorylation status of STAT3 (pSTAT3^Y705^), AKT (pAKT^S473^), ERK (pERK^T202/Y204^), and S6 (pS6^S235/S236^) was evaluated by immunoblot. Equal amounts of protein (20 µg) were added for each sample. **B**, The same NSCLC lines were plated as in A, but were treated with a fixed dose of ruxolitinib (1 µM) for the indicated time. Phospho-protein status was evaluated in 20 µg of protein per sample as in A. **C**, NCI-H1703 cells were infected with lentiviral vectors containing a control shRNA (NT, non-targeting) or one of two shRNAs targeted to human JAK2 (sh1 or sh2). Protein lysates were harvested and evaluated for JAK2 expression and STAT3 phosphorylation by immunoblot. Blots for total STAT3 and tubulin were included to demonstrate equal loading. **D**, Representative images of immunohistochemical stains for total JAK2, total STAT3 and phosphorylated STAT3 (pSTAT3^Y705^) performed on a tissue microarray containing 245 pathologically verified human NSCLC samples. Images were captured from staining performed serial sections of the same tumor samples. **E**, Summary of staining results for JAK2, STAT3 and pSTAT3^Y705^ on the NSCLC tissue microarray (*n* = 245). Note that all but one of the samples with positive nuclear pSTAT3^Y705^ staining (52/53, 98.1%) were also positive for JAK2.

Inflammatory cytokines that signal through gp130 primarily activate STAT proteins via non-receptor tyrosine kinases of the JAK family. This kinase family is composed of four members: JAK1, JAK2, JAK3 and Tyk2. Of these, only JAK2 is commonly expressed in cells of the epithelial lineage from which NSCLC arises [10], [28]. To test whether STAT3 is phosphorylated by JAK2, we treated the seven different NSCLC lines with the small molecule inhibitor ruxolitinib, which selectively targets both JAK1 and JAK2. Dose-response (Fig. 4A) and timecourse treatment (Fig. 4B) with ruxolitinib results in a rapid loss of STAT^Y705^ phosphorylation in all but one (NCI-H1993; not shown) of the NSCLC lines tested. Importantly, this drug had little to no effect on any other signaling pathways we tested, demonstrating its specificity for the JAK/STAT pathway relative to other mitogenic signaling cascades.

To further confirm that JAK2—and not JAK1—is responsible for STAT3^Y705^ phosphorylation in NSCLC, we used lentiviral shRNAs to specifically decrease expression of JAK2 *in vitro*. Depletion of JAK2 in NCI-H1703 cells—which demonstrate the highest levels of STAT3 and pSTAT3^Y705^ expression—results in a near complete loss of STAT3 phosphorylation comparable to ruxolitinib, supporting the hypothesis that JAK2 is the kinase primarily responsible for STAT3^Y705^ phosphorylation in NSCLC (Fig. 4C).

### JAK2 Expression Coincides with STAT3 Phosphorylation in Human NSCLC Samples

To address the importance of JAK2/STAT3 signaling in human tumor tissue samples, we performed immunohistochemistry (IHC) for JAK2, STAT3 and pSTAT3^Y705^ on a clinically annotated tissue microarray (Table S3) containing 245 stage I/II NSCLC samples (Fig. 4D). In the NSCLC samples analyzed, KRAS and EGFR mutations were present in 26.0% and 5.6% of patients, respectively.

The majority of tumors display positive staining for STAT3 (94%) and JAK2 (79%), while a smaller percentage display positive staining for pSTAT3^Y705^ (22%) (Fig. 4E). Importantly, all but one of the tumors that were scored as pSTAT3^Y705^ positive (*n* = 53) also scored positive for JAK2. Furthermore, staining for pSTAT3^Y705^ was positively correlated with the intensity of JAK2 staining (Pearson correlation coefficient 0.32; 95% Confidence Interval (CI) 0.20–0.43; *p* = 3.65×10^−07^), consistent with a key role for JAK2 in STAT3 activation. As would be expected for specific total/phospho-specific antibody pairs, positive pSTAT3^Y705^ staining was also positively correlated with the intensity of total STAT3 staining (Pearson correlation coefficient 0.37; 95% CI 0.25–0.47; *p* = 2.59×10^−09^).

We found no statistically significant correlations between histology, age and gender with the IHC staining or KRAS/EGFR mutational status, though KRAS mutational status was positively correlated with adenocarcinoma histology, similar to previous reports (Pearson chi-squared 12.38; *p-value* = 2.04×10^−03^) [29]. In addition, there were no significant correlations between KRAS or EGFR mutational status and IHC staining for any of the three proteins, nor were any of the IHC staining patterns significantly associated with relapse-free (RFS) or overall survival (OS). In line with the LACE (Lung Adjuvant Cisplatin Evaluation) meta-analysis, we found no significant correlation between EGFR or KRAS mutation status with RFS and OS [30]. The lack of correlation between prominent oncogenic mutations and STAT3 activation in human lung tumor samples further supports our *in vitro* finding that STAT3 phosphorylation is independent of key driver oncogenes, and is more likely an indicator of autocrine or stromal production of pro-inflammatory cytokines.

### JAK2 Inhibition Fails to Enhance Toxicity of Targeted Kinase Inhibitors

**Figure 5:**
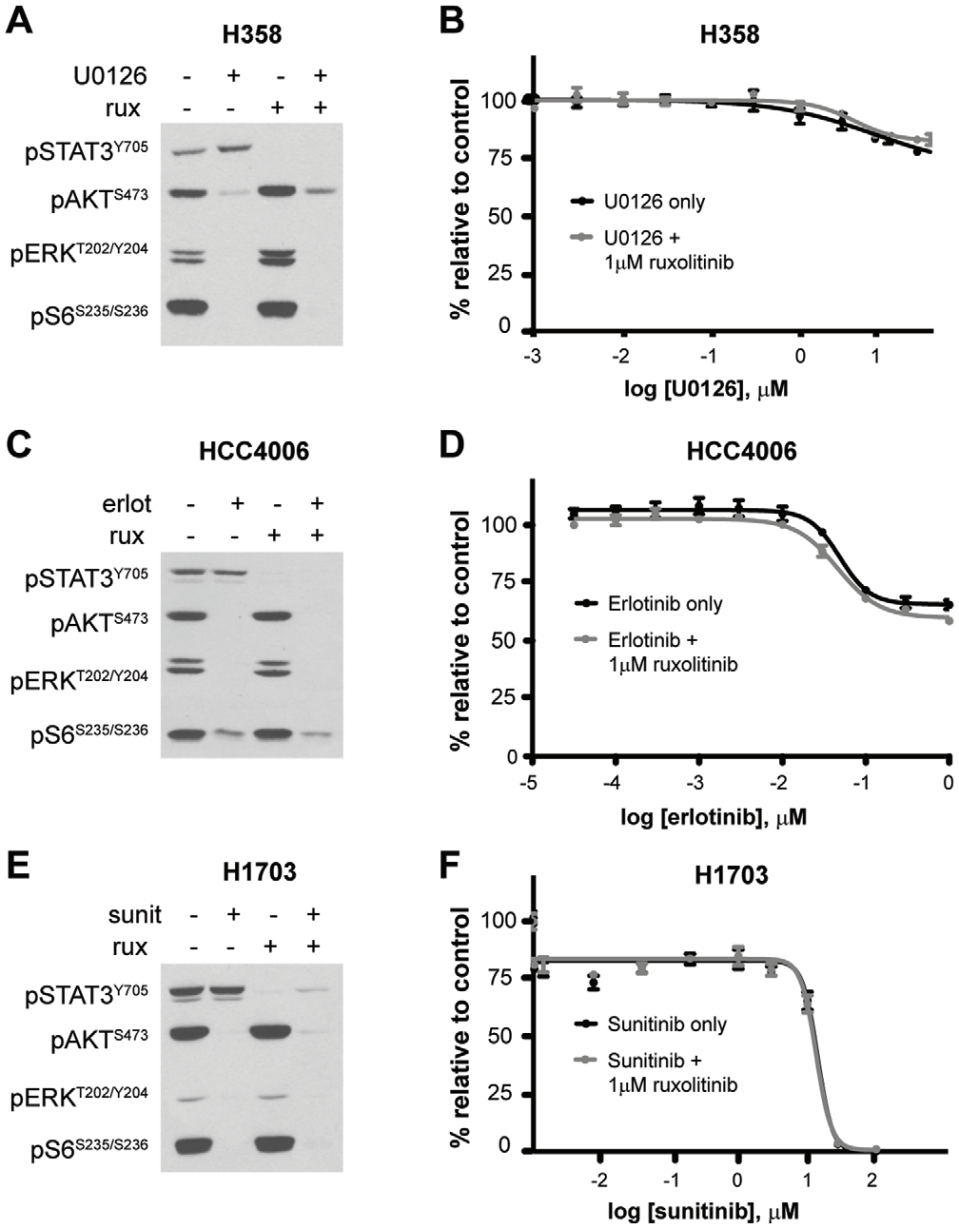
JAK2 inhibition with ruxolitinib does not affect kinase inhibitor toxicity in two-dimensional tissue culture. **A**, NCI-H358 cells (KRAS mutant) were plated at fixed density and treated for 24 hours with DMSO (−), U0126 (5.0 µM), ruxolitinib (1.0 µM) or both drugs. Protein lysates were harvested and the phosphorylation status of STAT3 (pSTAT3^Y705^), AKT (pAKT^S473^), ERK (pERK^T202/Y204^), and S6 (pS6^S235/S236^) was evaluated by immunoblot. Equal amounts of protein (20 µg) were added for each sample. **B**, NCI-H358 cells were treated in quadruplicate with a 3-fold serial dilution of the MEK1/2 inhibitor U0126 (range, 0–30 µM) +/− 1.0 µM ruxolitinib. Cell viability was measured 24 hours after treatment. Averaged values for each condition are shown as a percentage of vehicle-treated (DMSO) cells. Error bars indicate standard deviations of the four replicate values. **C**, HCC4006 cells (EGFR mutant) were plated at fixed density and treated for 24 hours with DMSO (−), erlotinib (1.0 µM), ruxolitinib (1.0 µM) or both drugs. Protein lysates were harvested and analyzed as in A. **D**, HCC4006 cells were treated in quadruplicate for 24 hours with a 3-fold serial dilution of the EGFR inhibitor erlotinib (range, 0–1.0 µM) +/− 1.0 µM ruxolitinib. Cell viability and percent survival was evaluated as in B. **E**, NCI-H1703 cells (PDGFR amplified/mutant) were plated at fixed density and treated for 24 hours with DMSO (−), sunitinib (1.0 µM), ruxolitinib (1.0 µM) or both drugs. Protein lysates were harvested and analyzed as in A. **F**, NCI-H1703 cells were treated in quadruplicate for 24 hours with a 3-fold serial dilution of the PDGFR inhibitor sunitinib (range, 0–100 µM) +/− 1.0 µM ruxolitinib. Cell viability and percent survival was evaluated as in B.

Because ruxolitinib and the targeted kinase inhibitors we tested appear to target distinct signaling pathways, we combined these agents together to achieve inhibition of both sets of effector proteins in each pathway. Combination of ruxolitinib with U0126, erlotinib or sunitinib effectively inhibited signaling to STAT3, AKT, ERK and S6, indicating that the JAK2, PI3K, MEK and mTOR pathways can be inhibited together using combinatorial drug treatment (Fig. 5A,C,E). To determine whether treatment with ruxolitinib enhances the efficacy of targeted kinase inhibitors *in vitro*, we treated three of the NSCLC lines with a three-fold serial dilution of their matched TKI in the presence or absence of 1 µM ruxolitinib. We found that ruxolitinib has little effect on the efficacy of any of these inhibitors in 24 hour viability (Fig. 5B,D,F) or 72 hour proliferation assays (Fig S2) in two-dimensional culture. Similarly, treatment of each of these cell lines with a three-fold serial dilution of ruxolitinib alone had no effect on cell viability or proliferation, even at doses that completely inhibit STAT3 phosphorylation (data not shown).

### JAK2/STAT3 Signaling Mediates Growth of NSCLC Cells in Soft Agar and In Vivo

**Figure 6:**
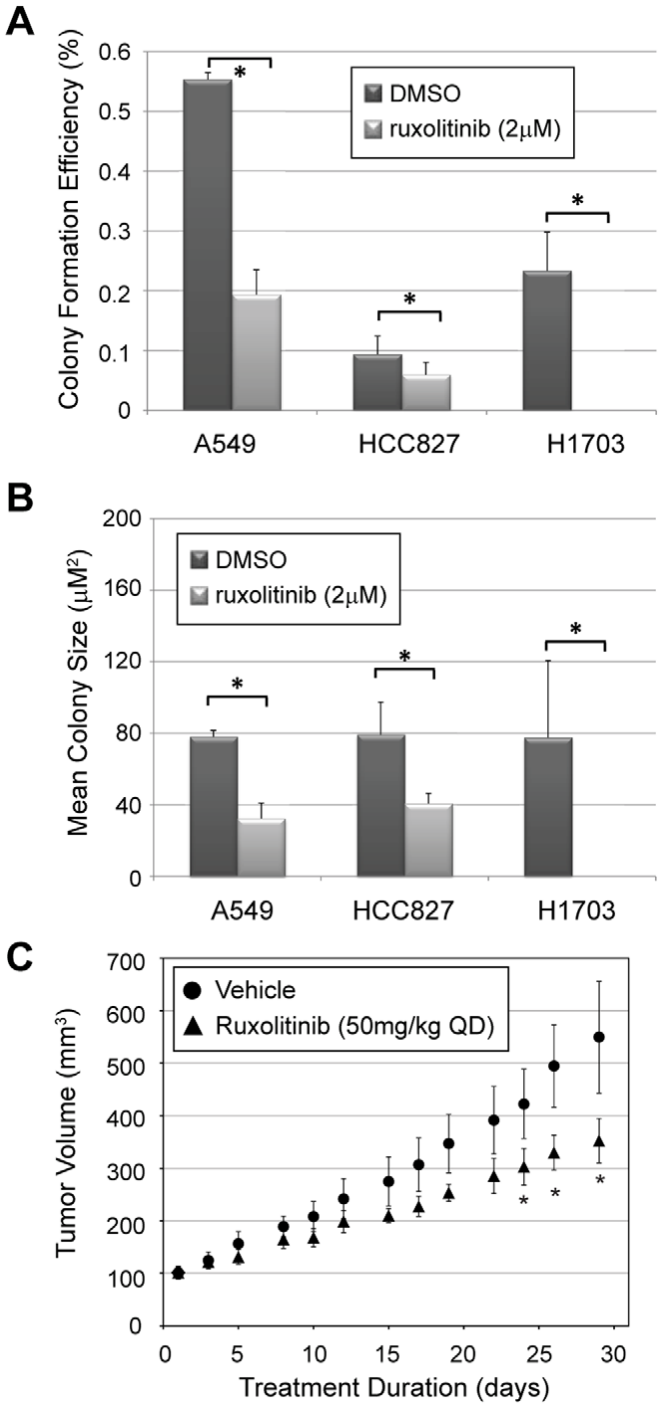
JAK2 inhibition with ruxolitinib inhibits NSCLC cell growth in soft agar and in xenograft assays. **A–B**, NSCLC cells were plated into a soft-agar suspension (5,000 cells/35 mm well) and overlaid with normal media containing 10% FBS +/− 2.0 µM ruxolitinib. After one month, agar plugs were fixed and stained with crystal violet to identify individual colonies. Digital images of each well were captured and analyzed for colony number and size. Average colony formation efficiency ([# colonies/# plated cells] ×100%, **A**) and mean colony size (area in µm^2^, **B**) for triplicate experiments are shown. Error bars indicate the standard deviation for triplicate wells. (*, *p-value*<0.05). **C**, HCC827 cells were xenografted into the flanks of nude mice. When tumors reached a volume of 100 mm^3^, mice were separated into two treatment arms (*n* = 6, each) and dosed daily by oral gavage with vehicle or the JAK2 inhibitor ruxolitinib (50 mg/kg). Tumor size was evaluated every other day. Animals were sacrificed when tumors reached 500 mm^3^ or after one month of treatment. Error bars indicate the standard error of tumor volume measurements. (*, *p-value*<0.05).

Similar results to those described above were recently obtained in RAS-transformed MCF10A cells, which do not require STAT3 for survival or proliferation in the context of two-dimensional tissue culture, but do require STAT3 for anchorage-independent growth [16]. To determine whether JAK2/STAT3 activity is required for growth of NSCLC lines in three-dimensional culture, we performed soft agar colony formation assays on each of the seven NSCLC in the presence of vehicle only (DMSO) or ruxolitinib (2 µM). Four of the seven lines we tested display anchorage independent growth by forming individual colonies in soft agar (A549, HCC827, NCI-H1703, NCI-H460). Continuous treatment of these cell lines with ruxolitinib decreases both the efficiency of colony formation (Fig. 6A) and the average size of colonies (Fig. 6B) in three of the four lines, indicating that JAK2/STAT3 signaling is important for anchorage-independent growth of specific subsets of NSCLC. The effect of ruxolitinib was particularly dramatic in NCI-H1703 cells, which failed to form any colonies at all in the presence of ruxolitinib. Notably, this cell line has the highest basal level of STAT3 phosphorylation of all the cell lines we tested (Fig. 2, 3).

To further validate the effect of JAK2 inhibition on tumor growth *in vivo*, we performed xenograft assays with HCC-827 lung adenocarcinoma cells, which are driven by an activating mutation (E746-A750 deletion) in EGFR [31]. We injected 5×10^6^ cells into the flanks of nude mice and allowed tumors to reach a starting volume of 100 mm^3^. The mice were then separated into two groups of six mice each and treated daily with vehicle (0.5% hydroxypropylmethylcellulose/0.2% Tween 80 in PBS) or ruxolitinib (50 mg/kg) by oral gavage. We found that ruxolitinib treatment slowed tumor growth *in vivo*, decreasing tumor volume by 36% (vehicle, 549.6±260.2 mm^3^ vs. ruxolitinib, 352.8±103.7 mm^3^) over 30 days of treatment (Fig. 6C). These data are consistent with the soft agar growth assays, and confirm the importance of JAK2/STAT3 signaling in the growth of NSCLC tumors.

## Discussion

The JAK/STAT signaling pathway has long been associated with hematologic malignancies, particularly those of the myeloid lineage. An essential role for JAK2/STAT3 has been most clearly identified in myeloproliferative disorders (polycythemia vera, essential thrombocythemia, idiopathic myelofibrosis) and several different myeloid malignancies (atypical CML, CMML, megakaryocytic AML, JMML), all of which bear a high proportion of somatic JAK2 mutations (i.e. V617F) that lead to constitutive kinase activation [32]. The strong association of JAK2 mutations with these cancers—as well as a wealth of preclinical validation—have led to the development of several JAK inhibitors, many of which are already in various stages of clinical testing [33]. Most notable among these drugs is the JAK1/2 selective inhibitor ruxolitinib (INCB018424), which has shown promising results and relatively low toxicity in phase I/II clinical testing of patients with primary myelofibrosis [34]. Use of this inhibitor in JAK2-transformed cells leads to rapid dephosphorylation of STAT3 concomitant with growth arrest and death of these cells [35].

While emerging studies continue to implicate STAT3 as an important oncogene in solid tumors, it is still unclear precisely how STAT3 becomes activated in this context [10], [11]. Phosphorylation of STAT3 at Y705 is both positively and negatively regulated by several different mechanisms, several of which have direct relevance to NSCLC. Negative regulators of STAT3 in particular are common targets of mutation, silencing or deletion in NSCLC. Direct dephosphorylation of STAT3 is achieved by the phosphatase PTPRD, which is commonly mutated or epigenetically silenced in the majority of NSCLC [36]. STAT3 activity is also regulated by a negative feedback loop involving SOCS3, a STAT3 target gene that is transcriptionally activated following STAT3 activation [37]. SOCS3 binds to activated tyrosine kinases, thereby limiting their access to substrates including STAT3. This effectively limits the positive input to STAT3, and allows for effective dephosphorylation and inactivation. Notably, SOCS3 has also been shown to be epigenetically silenced in NSCLC, suggesting a selective role for STAT3 activation in this disease.

Positive regulators of Y705 phosphorylation include the Janus kinase (JAK) and SRC family of non-receptor tyrosine kinases, both of which are commonly hyperactivated in solid malignancies. Unlike hematologic malignancies, however, activating mutations to JAK family kinases are rarely found in epithelial-derived solid tumors [38], [39]. Instead, these kinases appear to be activated by upstream signal transduction pathways induced by mutated growth factor receptors or inflammatory cytokines, particularly those of the IL-6 family [24], [40], [41], [42], [43]. The relative contributions of each of these different stimuli to STAT3 activation in NSCLC is currently unclear, and poses an important problem for pharmacologic management of this disease.

In this study we show that STAT3 is predominantly activated by JAK2-dependent phosphorylation of Y705 in NSCLC lines that are transformed by diverse driver mutations. We also demonstrate that inhibition of MEK, EGFR, MET, or PDGFRA is unable prevent activation of STAT3 in NSCLC, suggesting that these hyperactive growth factor signaling pathways do not play a major role in the constitutive activation of STAT3. As such, our data imply that pharmacologic therapies targeting these pathways in NSCLC are unlikely to impact STAT3 or any of the cellular phenotypes that it transcriptionally regulates. Because STAT3 signaling has been implicated in therapeutic resistance to several different cancer treatments, ongoing efforts to target receptor tyrosine kinase signaling networks in NSCLC would likely be improved by combination with ruxolitinib or other JAK family inhibitors [14], [15], [44].

Previous studies have clearly implicated the cytokine IL-6 as a key activator of JAK2 in NSCLC [14], [24]. While three of the seven cell lines we examined confirm this finding, our data also suggest that IL-6 is not the only cytokine involved in this process. The other four lines we analyzed fail to produce detectable levels of soluble IL-6, but remain sensitive to gp130 neutralization, suggesting that other IL-6 family ligands might be involved. This finding implies that therapeutic neutralization of IL-6 itself with humanized monoclonal antibodies—which has been developed for other auto-inflammatory conditions—is unlikely to be as effective as inhibiting the downstream effectors of all IL-6 family ligands, which include the gp130 receptor and JAK family kinases. As such, future combinatorial approaches should focus on therapies that target these molecules in addition to key “driver oncogenes” such as KRAS and EGFR.

## Materials and Methods

### Cell Culture

All seven NSCLC lines (HCC827, HCC4006, A549, NCI-H358, NCI-H460, NCI-H1703, NCI-H1993) were obtained from ATCC. Cells were grown in normal tissue culture-treated flasks in RPMI-1640 media containing 10% fetal bovine serum (FBS) under standard growth conditions of 5% CO_2_. Cells were passaged at 85–90% confluency every 3–4 days to maintain continuous logarithmic growth. Drug treatments with erlotinib (LC Labs, Woburn, MA), U0126 (Calbiochem), sunitinib (LC Labs), crizotinib (Selleck Chemicals, Houston, TX), and ruxolitinib (LC Labs) were performed as described in the text. All drugs were resuspended in DMSO prior to dilution in cell culture media. Stimulation with recombinant CLCF1/CRLF1 heterodimeric ligand (R&D Systems, Minneapolis, MN) was performed in serum-free RPMI-1640 media using 5 ng/mL ligand concentration.

### Antibodies

Phospho-specific antibodies for pEGFR^Y1068^, pSTAT3^Y705^, pAKT^S7431^, pERK^T202/Y204^ and pS6^S235/S236^ as well as the total STAT3 and JAK2 antibodies were obtained from Cell Signaling Technologies (Danvers, MA). The mouse monoclonal antibody to tubulin was obtained from Sigma-Aldrich (St. Louis, MO). The neutralizing mouse monoclonal to gp130 was obtained from R&D Systems. The mouse monoclonal and rabbit polyclonal antibodies used for the CLCF1 sandwich ELISA assay were obtained from R&D Systems and Abcam (Cambridge, MA) respectively.

### Immunoblotting

Cells grown under normal culture conditions were washed with cold PBS and harvested on ice in cold pH 7.5 lysis buffer (20 mM Tris-HCl , 150 mM NaCl, 1 mM Na_2_EDTA, 1 mM EGTA, 2.5 mM sodium pyrophosphate, 1 mM β-glycerophosphate, 50 mM sodium fluoride, 1 mM Na_3_VO_4_, 1% Triton-X100, 1 mM DTT) supplemented with protease inhibitor cocktail (Sigma-Aldrich, St. Louis, MO). Soluble protein from lysates was quantified by Bradford assay (Bio-Rad, Hercules, CA). After normalization of concentration, samples were diluted with Laemmli buffer and denatured by boiling. Sample were then separated on Tris-glycine polyacrylamide gels and transferred overnight to nitrocellulose or nitrocellulose membranes in a wet transfer apparatus (Hoefer, Holliston, MA). Membranes were blocked in 3% non-fat dry milk in Tris-buffered saline/0.1% Tween (TBS-T) and probed with primary antibodies overnight at 4°C. After washing in TBS-T buffer and incubation with a horseradish peroxidase-coupled secondary antibody, membranes were incubated in enhanced chemiluminescent reagent, exposed to film and developed for signal using an *X-omat* processing machine (Kodak, Rochester, NY).

### Phospho-protein Array Analysis

Cell culture lysates obtained as indicated above were analyzed using the Pathscan RTK Signaling Antibody Array Kit according to the manufacturer recommended protocol (Cell Signaling, Danvers, MA). Briefly, 250 µg of protein lysate from each cell line was applied to a pre-blocked antibody array and incubated overnight at 4°C with constant rocking. The arrays were washed with PBS/0.05% Tween buffer and serially incubated for 1 hour with the biotinylated detection antibody cocktail and DyLight-680-conjugated streptavidin. After further washing, the arrays were dried and imaged using a laser microarray scanner (Tecan, Männedorf, Switzerland). Duplicate spot intensities were quantified from each array image using the QuantityOne software package (BioRad, Hercules, CA).

### Cyotokine Measurements

Conditioned media was collected from all seven cell lines 48 hours after plating in serum-free media. The samples were submitted to Rules-Based Medicine (RBM, Austin, TX) for bead-based immunodetection of 48 different cytokines including IL-6 (Inflammatory Cytokine Panel) (see Table S2 for complete list). Replicate samples of conditioned media were concentrated with 10 kilodalton cutoff filtration vials (Millipore, Billerica, MA) and assayed for CLCF1 using a sandwich ELISA protocol. Briefly, a 96-well plate was coated with rabbit polyclonal anti-CLCF1/NNT1 (Abcam, diluted 1∶500 in PBS) overnight at 4°C. After washing 4 times with TBS/0.05% TritonX-100 (TBST), plates were coated with 200 µL conditioned media. Antigen was allowed to adhere overnight at at 4°C. Plates were then washed as above and incubated for two hours with mouse monoclonal anti-CLCF1 (R&D Systems, diluted 1∶500 in TBST/0.1% BSA). Plates were washed again and incubated an additional hour with HRP-conjugated goat anti-mouse secondary antibody. After a final round of washing, plates were incubated with tetramethylbenzidine (TMB) substrate (Cell Signaling) for 5 minutes, quenched with 0.16 M sulfuric acid and measured for absorbance at 450 nm using a multiplate reader (Molecular Devices, Sunnydale, CA). Concentrations were normalized to a standard curve generated with recombinant CLCF1 standard (R&D Systems) diluted in serum-free media (see Fig S1B).

### Quantitative RT-PCR

Total mRNA was harvested from cells grown under normal conditions using the RNeasy miniprep kit according to manufacturer protocol (Qiagen, Valencia, CA). RNA was quantified by UV-spectrophotometry and normalized for input of 1.0 µg of RNA into each cDNA synthesis reaction. Template cDNA was synthesized using the iScript-Select kit and poly-dT primers (Bio-Rad, Hercules, CA) according to standard manufacturer protocol with a 90 minute extension phase to optimize synthesis of long transcripts. The products of each cDNA synthesis reaction were diluted 1∶5 in Tris-EDTA buffer and used as template for quantitative PCR. PCR reactions for each sample contained 10 µL of 2× SYBR green reaction mix (Roche Applied Science, Indianapolis, IN), 5 µL of template cDNA, 1.0 µM primers (IDT, Coralville, IA) and sterile deionized water to a final 20 µL volume. Reactions were performed on a 7500 Real Time thermocycler (Applied Biosystems, Foster City, CA) according to standard protocol with an added melting curve phase to ensure a single PCR product was detected in each well. All reactions to quantify IL-6 family ligand mRNAs were performed in triplicate and normalized to averaged triplicate measurements of the housekeeping gene *RPL13A*. Primers for these genes are indicated in Table S4.

### Immunofluorescent Staining and Microscopy

Cells were seeded to coverslips and allowed to adhere and grow for 48 hours. Cells were then fixed with 4% paraformaldehyde and permeabilized with 0.2% TritonX-100 in PBS. After blocking with 5% normal goat serum in PBS, the coverslips were incubated at 4°C overnight with a 1∶100 dilution mouse monoclonal anti-STAT3 antibody (Cell Signaling Technology). After washing in PBS/0.02% TritonX-100, coverslips were incubated for one hour with AlexaFluor-488 coupled anti-mouse secondary antibodies. After a final round of washing, cells were co-stained with DAPI to detect nuclei and coverslips were mounted on glass slides with anti-fade gel mounting medium. Pictures were obtained using an LSM-510 confocal microscope and LSM Image Browser software (Zeiss, Stuttgart, Germany).

### Production of Stable shRNA-Expressing Cell Lines

Stable knockdown cell lines were created by infecting cells with pLK0.1-based lentiviral particles containing shRNAs targeted to JAK2 (Sigma-Aldrich, St. Louis, MO) (JAK2-shRNA-1 [#TRCN0000003179], JAK2-shRNA-2 [#TRCN0000003180]). Forty-eight hours post-infection, cells were passaged and selected with 2.0 µg/mL puromycin for an additional 72–96 hours prior to analysis to eliminate uninfected cells. Stable lines were routinely used for all assays within 1 week of selection to eliminate artifacts caused by random selection for shRNA inactivation.

### Tissue Acquisition and Tissue Microarray Construction

All lung tumor tissues analyzed in this study were retrospectively obtained after prior approval of the Scottsdale Healthcare IRB under Exemption 4 of Title 45 Code of Federal Regulations (CFR) concerning retrospective study of existing data. Patient consent is not required under this exemption and was not obtained for this study, as Title 45 CFR Part 46 does not apply. The clinical information associated with these specimens is not individually identifiable, and was collected in such a manner that subjects cannot be identified either directly or indirectly through identifiers linked to the subjects. Tumor tissues were from patients diagnosed between 2001 and 2007 with stage I–II NSCLC according to AJCC 7^th^ edition criteria [45] who received care and follow-up at Scottsdale Healthcare (SHC, Scottsdale, AZ). Tumor tissues were collected from patients that underwent either surgical resection or definitive radiation therapy and did not have a concomitant diagnosis of malignancy within the preceding five years. Pathological confirmation of NSCLC and histologic subtype was rendered by a SHC board-certified pathologist at the time of diagnosis. Confirmation of staging, collection and database maintenance of clinical characteristics such as age at diagnosis, gender, histology, stage, smoking history, and relapse-free and overall survival was conducted by a board-certified medical oncologist (GJW). The presence of baseline medical co-morbidities including: diabetes, hypertension, cardiovascular disease (CAD), lung disease [chronic obstructive pulmonary disease (COPD) or emphysema], thrombotic event (deep vein thrombosis or pulmonary embolism), peripheral vascular disease (PVD), and hyperlipidemia were also collected, where available. Hematoxylin and eosin (H&E) stained slides of each tumor were reviewed by a board-certified pathologist at TGen to identify representative tumor regions in each sample. Two tissue cores (1 mm in diameter) were taken from distinct regions of the primary tumor from each case using a ATA100 tissue microarrayer (Chemicon, Temecula, CA).

### Immunohistochemistry

Freshly cut 5 micron sections from tissue microarray slides of human lung tumors were deparaffinized with xylenes and rehydrated through a series of graded ethanols. Subsequent staining steps were performed on a Discovery XT automated immunostainer (Ventana, Tucson, AZ). Antigen retrieval on all tissues was performed by heating slides to 100°C in Tris-EDTA-borate buffer, and then allowing them to cool gradually to room temperature. Tissues were incubated with primary antibody (anti-JAK2, 1∶200; anti-STAT3, 1∶200; or pSTAT3^Y705^, 1∶100) for 32 minutes at room temperature and then processed with the UltraMAP biotin-free rabbit secondary antibody kit (Ventana). After immunostaining, all tissues were counterstained with hematoxylin and coverslipped using a Symphony automated stainer (Ventana). Stained tissues were scored using the HSCORE method, which is a composite of percent stained cells multiplied by an intensity score (value of 0, 1, 2 or 3), allowing for a range of 0–300 for each individual tissue [46]. For STAT3 and JAK2, a score >100 was considered positive; for pSTAT3^Y705^, a score >40 was considered positive.

### Statistical Analysis

All statistical analyses were performed using the R statistical package (version 2.10.1). The relationship between two-class discretized IHC values for individual antigens and histological classes was assessed using Fisher's exact test or the Chi-squared test. Correlation between continuous variables was assessed using the test for correlation between paired samples, employing Pearson's correlation coefficient. Survival analysis was performed for right censored patient survival using Cox proportional hazards regression models as implemented in the R survival package (version 2.35–8). Models using continuous and two-class discretized IHC values, as well as KRAS or EGFR mutation status as predictors, were independently tested. Histological classes were defined as adenocarcinoma (“Adenocarcinoma”, “mixed adeno”, “Mucinous adenocarcinoma”, “Papillary adenocarcinoma”, “bronchioloalveolar carcinoma [BAC]”), Squamous (“Squamous”) or other (“Large cell carcinoma”, “NSCLC”).

### Cell Proliferation & Viability Analyses

Proliferation and survival curves were generated using the Cell Titer Glo assay (Promega, Madison, WI). Luminescent values for each condition were measured in quadruplicate wells and normalized to media only control wells using an Envision multiplate reader (PerkinElmer, Waltham, MA). Viability was calculated as a percentage of untreated cells from the same line 24 hours post-treatment. Proliferation was calculated as a percentage of untreated cells from the same line 72 hours after drug treatment. Error bars represent the standard deviation of triplicate values for each assay. Values were fit to survival and proliferation curves using the Prism 5 software package (GraphPad Software, La Jolla, CA).

### Soft Agar Colony Formation Assays

Cell lines were suspended in RPMI-1640 media containing 0.35% agarose at 40°C at a density of 5,000 cells per 1.5 mL media. This suspension was plated on top of a 1.5 mL plug of pre-solidified RPMI-1640 media containing 0.5% agarose in a 6-well plate. After allowing the cell suspension to solidify for overnight under normal TC conditions, each agar plug was overlaid with 2 mL of RPMI-1640 media containing the indicated drug. Media was changed three times per week on alternate days to maintain adequate nutrient and drug concentrations until visible colonies could be observed in the DMSO-treated wells. Upon the completion of the assay, each agar plug was fixed and stained overnight in a PBS solution containing 1% formaldehyde and 0.005% crystal violet. Agar plugs were destained by washing thoroughly with PBS and imaged using a digital scanner (Hewlett-Packard, Palo Alto, CA). Images were quantified for colony number and size using the NIS Elements software package (Nikon, Melville, NY). All assays were performed in triplicate. Error bars represent standard deviation of values for each condition. Statistical significance was determined by unpaired student t-test.

### Xenograft Assays

Xenograft studies were performed at The Center for Translational Drug Development (TD2, Scottsdale, AZ) in compliance with the Translational Genomics Research Institute Institutional Animal Care and Use Committee (IACUC) under project number TD2724. HCC827 cells were suspended in sterile, serum-free RPMI-1640 growth medium at a concentration of 25×10^6^ cells/mL. A volume of 0.2 mL (5×10^6^ cells) was inoculated into the right flank of athymic nude mice, which were monitored for tumor growth at the site of inoculation by palpation every other day until tumors became palpable. Upon palpability, tumor size was measured every other day using calipers and the tumor volume was calculated using the formula (length × width^2^ ×0.52). When mean tumor volume reached 100 mm^3^, the mice were randomized into two different treatment arms with six mice per arm. A stock solution of ruxolitinib was prepared in ethanol and suspended in vehicle (0.5% hydroxypropyl-methylcellulose/0.2% Tween 80 in PBS) prior to treatment. Mice were treated daily by oral gavage with vehicle or ruxolitinib (50 mg/kg). The primary endpoints for the study were one month (30 days) of drug treatment or achievement of 500 mm^3^ volume.

## Supporting Information

Figure S1 **Quantification of IL-6 Family Ligand Expression.** **A**, Quantitative RT-PCR was performed on cDNA samples obtained from each of the seven indicated NSCLC cell lines. Percent expression was normalized to the housekeeping gene *RPL13A*. Error bars represent standard deviations of triplicate measurements. **B**, Sandwich ELISA standard curve of CLCF1 ligand determined using a two-fold serial dilution of recombinant CLCF1 in serum-free media. Absorbance at 450 nM is plotted against the concentration of CLCLF1. **C**, Secreted CLCF1 levels were measured in 48 hour conditioned media samples by sandwich ELISA. Values are duplicate measurements from each cell line. *nd*, not detected. **D**, The indicated cell lines were retained in basal media containing 10% fetal bovine serum (basal), or serum-starved for one hour prior to treatment +/− recombinant CLCF1/CRLF1 (5 ng/mL). The expression level and phosphorylation status of STAT3 (pSTAT3^Y705^) were evaluated by immunoblot, along with β-actin as a loading control. The neuroblastoma cell line SH-SY5Y was included as a positive control to demonstrate bioactivity of the recombinant CLCF1/CRLF1 ligand.
(TIF)

Figure S2 **JAK2 inhibition with ruxolitinib does not affect proliferation of NSCLC lines in two-dimensional tissue culture.** **A**, NCI-H358 cells were treated in quadruplicate with a 3-fold serial dilution of the MEK1/2 inhibitor U0126 (range, 0–30 µM) +/− 1.0 µM ruxolitinib. Cell number was measured at 72 hours after treatment and normalized to untreated controls. Averaged values for each condition are shown as a percentage of vehicle-treated (DMSO) cells. Error bars indicate standard deviations of the four replicate values. **B**, HCC4006 cells were treated in quadruplicate for 72 hours with a 3-fold serial dilution of the EGFR inhibitor erlotinib (range, 0–1.0 µM) +/− 1.0 µM ruxolitinib. Cell number and percent proliferation were evaluated as in A. **C**, NCI-H1703 cells were treated in quadruplicate for 24 hours with a 3-fold serial dilution of the PDGFR inhibitor sunitinib (range, 0–100 µM) +/− 1.0 µM ruxolitinib. Cell number and percent proliferation were evaluated as in A.
(TIF)

Table S1 **NSCLC Cell Line Mutational Profile.** Mutational status of common oncogenes and tumor suppressors in the seven NSCLC lines used for this study were obtained from the Catalogue of Somatic Mutations in Cancer (COSMIC) database [47].
(PDF)

Table S2 **NSCLC Cell Line Cytokine Measurements.** Duplicate measurements of 46 different inflammatory cytokines in conditioned media from the seven NSCLC cell lines used in this study was performed using a commercial bead-based immunoassay (Human InflammationMAP_v1.0). For each cytokine, the least detectable dose (LDD) was determined as the mean +3 standard deviations of 20 blank readings. Results below the LDD are more variable than results above the LDD. The value <LOW> reflects samples not measurable on the standard curve.
(PDF)

Table S3 **Patient disease characteristics and outcome statistics.** Key clinical, histological and treatment features, as well as outcome statistics, of the patient population represented on our tissue microarray are indicated. Mutational status for KRAS and EGFR are also shown for this population.
(PDF)

Table S4 **Primers for IL-6 Family q-RT-PCR.** Oligonucleotide sequences of each primer pair (forward and reverse, 5′-to-3′) used in quantitative RT-PCR detection of IL-6 family ligands are shown. Each pair was validated to produce a single PCR fragment of the expected length by gel electrophoresis.
(PDF)
