# Supplementary figures and images for: STAT3 Is Activated by JAK2 Independent of Key Oncogenic Driver Mutations in Non-Small Cell Lung Carcinoma
Source: PLoS One. 2012 Feb 2;7(2):e30820. doi: 10.1371/journal.pone.0030820 (PMC3271110; doi:10.1371/journal.pone.0030820)

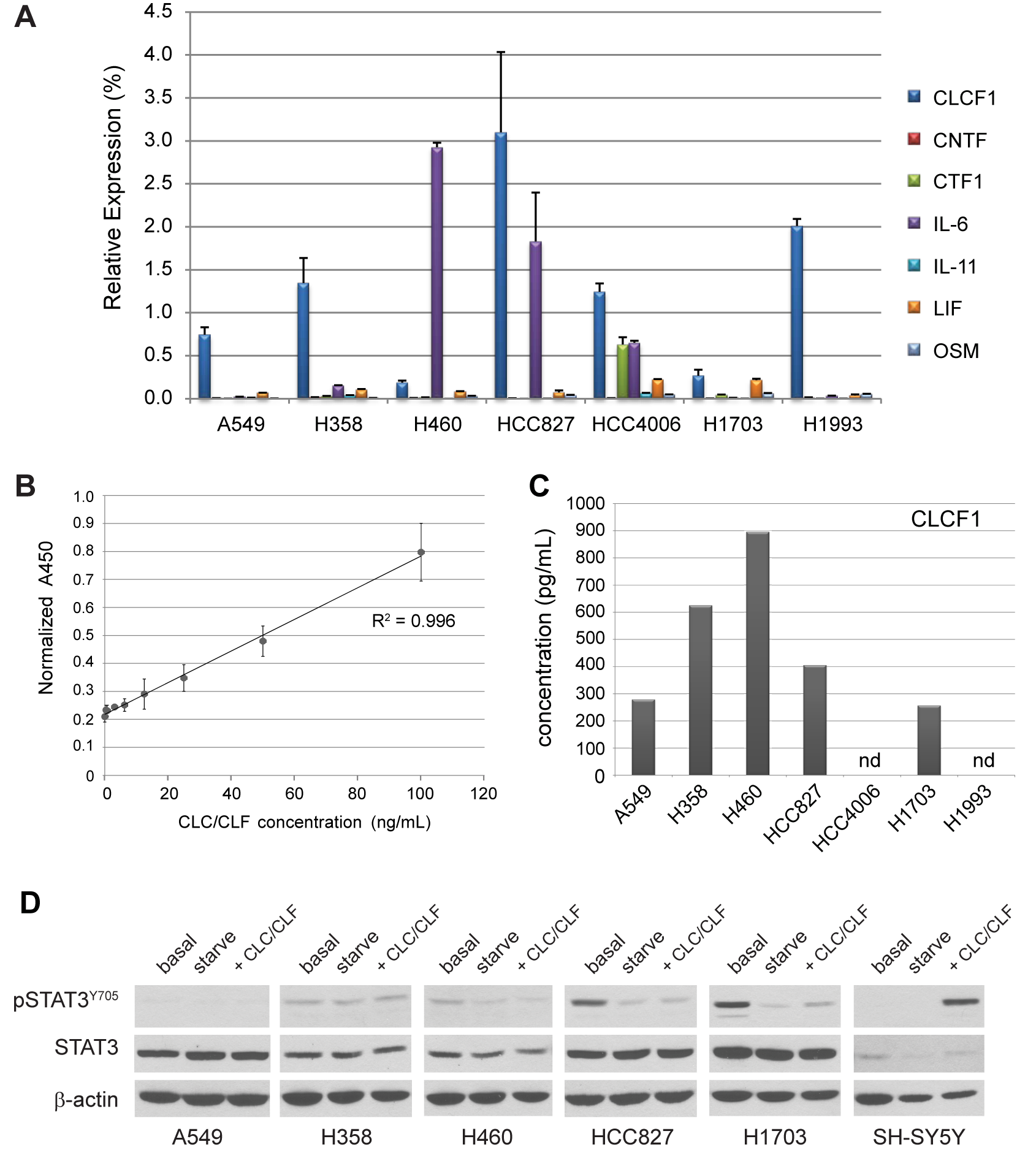

Supplement: Figure S1 — Quantification of IL-6 Family Ligand Expression. A, Quantitative RT-PCR was performed on cDNA samples obtained from each of the seven indicated NSCLC cell lines. Percent expression was normalized to the housekeeping gene RPL13A. Error bars represent standard deviations of triplicate measurements. B, Sandwich ELISA standard curve of CLCF1 ligand determined using a two-fold serial dilution of recombinant CLCF1 in serum-free media. Absorbance at 450 nM is plotted against the concentration of CLCLF1. C, Secreted CLCF1 levels were measured in 48 hour conditioned media samples by sandwich ELISA. Values are duplicate measurements from each cell line. nd, not detected. D, The indicated cell lines were retained in basal media containing 10% fetal bovine serum (basal), or serum-starved for one hour prior to treatment +/− recombinant CLCF1/CRLF1 (5 ng/mL). The expression level and phosphorylation status of STAT3 (pSTAT3Y705) were evaluated by immunoblot, along with β-actin as a loading control. The neuroblastoma cell line SH-SY5Y was included as a positive control to demonstrate bioactivity of the recombinant CLCF1/CRLF1 ligand. (TIF) [file pone.0030820.s001.tif]

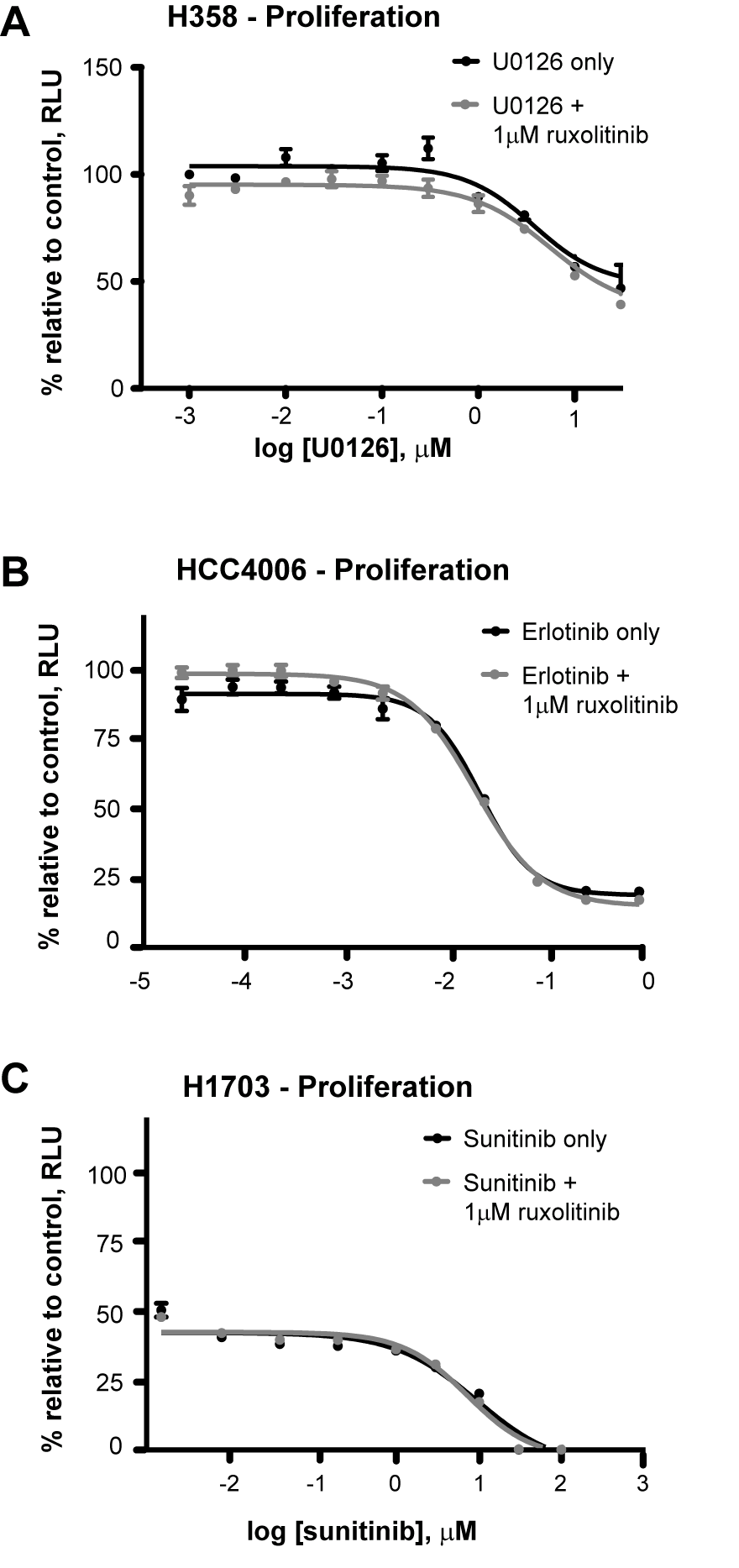

Supplement: Figure S2 — JAK2 inhibition with ruxolitinib does not affect proliferation of NSCLC lines in two-dimensional tissue culture. A, NCI-H358 cells were treated in quadruplicate with a 3-fold serial dilution of the MEK1/2 inhibitor U0126 (range, 0–30 µM) +/− 1.0 µM ruxolitinib. Cell number was measured at 72 hours after treatment and normalized to untreated controls. Averaged values for each condition are shown as a percentage of vehicle-treated (DMSO) cells. Error bars indicate standard deviations of the four replicate values. B, HCC4006 cells were treated in quadruplicate for 72 hours with a 3-fold serial dilution of the EGFR inhibitor erlotinib (range, 0–1.0 µM) +/− 1.0 µM ruxolitinib. Cell number and percent proliferation were evaluated as in A. C, NCI-H1703 cells were treated in quadruplicate for 24 hours with a 3-fold serial dilution of the PDGFR inhibitor sunitinib (range, 0–100 µM) +/− 1.0 µM ruxolitinib. Cell number and percent proliferation were evaluated as in A. (TIF) [file pone.0030820.s002.tif]
