# Supplementary material for: STAT3 Is Activated by JAK2 Independent of Key Oncogenic Driver Mutations in Non-Small Cell Lung Carcinoma
Source: PLoS One. 2012 Feb 2;7(2):e30820. doi: 10.1371/journal.pone.0030820 (PMC3271110; doi:10.1371/journal.pone.0030820)
Supplement: Table S1 — NSCLC Cell Line Mutational Profile. Mutational status of common oncogenes and tumor suppressors in the seven NSCLC lines used for this study were obtained from the Catalogue of Somatic Mutations in Cancer (COSMIC) database [47]. (PDF) [file pone.0030820.s003.pdf]

Cell line	EGFR	KRAS	STK11	CDKN2A	PIK3CA	TP53	PDGFRA	MET	CTNNB1
**HCC4006**	L747-A750 del	-	-						
**HCC827**	E746-A750 del	-	-					amp	
**A549**	-	G12S	Q37*	del	-	-		-	-
**NCI-H358**	-	G12C	-	-	-	-			T75A
**NCI-H460**	-	Q61H	Q37*	del	E545K	-		-	-
**NCI-H1703**	-	-	-	D84V	-	c.919-1G>T	amp;S478P		
**NCI-H1993**	-	-	E199*	del	-	C242W		amp	-

Supplemental Table S1. NSCLC Cell Line Mutational Profile.
Mutational status of common oncogenes and tumor suppressors in the seven NSCLC lines used for this study were obtained from the Catalogue of Somatic Mutations in Cancer (COSMIC) database [47].
