# Supplementary material for: STAT3 Is Activated by JAK2 Independent of Key Oncogenic Driver Mutations in Non-Small Cell Lung Carcinoma
Source: PLoS One. 2012 Feb 2;7(2):e30820. doi: 10.1371/journal.pone.0030820 (PMC3271110; doi:10.1371/journal.pone.0030820)
Supplement: Table S2 — NSCLC Cell Line Cytokine Measurements. Duplicate measurements of 46 different inflammatory cytokines in conditioned media from the seven NSCLC cell lines used in this study was performed using a commercial bead-based immunoassay (Human InflammationMAP_v1.0). For each cytokine, the least detectable dose (LDD) was determined as the mean +3 standard deviations of 20 blank readings. Results below the LDD are more variable than results above the LDD. The value reflects samples not measurable on the standard curve. (PDF) [file pone.0030820.s004.pdf]

Group Designation Human NSCLC Panel
Test Profile Human InflammationMAP™ 1.0

Analytes	Alpha-2-Macroglobulin (A2Macro)	Alpha-1-Antitrypsin (AAT)	Beta-2-Microglobulin (B2M)	Brain-Derived Neurotrophic Factor (BDNF)	Complement C3 (C3)	C-Reactive Protein (CRP)	Eotaxin-1	Factor VII	Fibrinogen	Ferritin (FRTN)	Granulocyte-Macrophage Colony-Stimulating Factor (GM-CSF)
**Units**	mg/mL	mg/mL	ug/mL	ng/mL	mg/mL	ug/mL	pg/mL	ng/mL	mg/mL	ng/mL	pg/mL
**Least Detectable Dose**	0.00010	3.6E-08	0.00029	0.0039	2.3E-08	1.6E-06	20	0.56	7.0E-08	0.012	1.8
**RBM Low Serum Range**	0.40	0.86	1.3	0.27	0.89	0.18		129		8.8	
**RBM High Serum Range**	7.9	2.1	4.4	31	2.5	69	218	682	0.030	631	39
**<u>Samples</u>**											
**A549**	3.7E-05	2.4E-07	0.026	0.0095	1.5E-05	<LOW>	93	0.61	3.6E-06	0.68	<LOW>
**HCC4006**	5.0E-05	3.0E-05	0.11	0.0055	3.0E-08	<LOW>	78	1.2	1.2E-07	0.076	29
**HCC827**	5.5E-05	5.0E-05	0.072	0.021	1.5E-05	3.6E-06	129	2.4	1.7E-06	0.083	112
**H358**	6.9E-05	<LOW>	0.065	0.015	1.4E-07	<LOW>	130	2.9	7.4E-08	0.21	5.7
**H460**	7.0E-05	<LOW>	0.12	0.065	8.4E-07	<LOW>	119	0.87	7.4E-08	1.3	0.75
**H1703**	6.7E-05	<LOW>	0.014	<LOW>	<LOW>	<LOW>	89	0.61	<LOW>	0.30	<LOW>
**H1993**	8.0E-05	<LOW>	0.066	0.039	9.5E-06	<LOW>	128	2.4	<LOW>	0.074	0.75

Supplemental Table S2. NSCLC Cell Line Cytokine Measurements.
Duplicate measurements of 46 different inflammatory cytokines in conditioned media from the seven NSCLC cell lines used in this study was performed using a commercial bead-based immunoassay (Human InflammationMAP_v1.0). For each cytokine, the least detectable dose (LDD) was determined as the mean +3 standard deviations of 20 blank readings. Results below the LDD are more variable than results above the LDD. The value <LOW> reflects samples not measurable on the standard curve.

Haptoglobin	Intercellular Adhesion Molecule 1 (ICAM-1)	Interferon gamma (IFN-gamma)	Interleukin-1 alpha (IL-1 alpha)	Interleukin-1 beta (IL-1 beta)	Interleukin-10 (IL-10)	Interleukin-12 Subunit p40 (IL-12p40)	Interleukin-12 Subunit p70 (IL-12p70)	Interleukin-15 (IL-15)	Interleukin-17 (IL-17)	Interleukin-18 (IL-18)	Interleukin-1 receptor antagonist (IL-1ra)	Interleukin-2 (IL-2)
mg/mL	ng/mL	pg/mL	ng/mL	pg/mL	pg/mL	ng/mL	pg/mL	ng/mL	pg/mL	pg/mL	pg/mL	pg/mL
9.8E-08	0.48	0.38	0.0015	0.36	0.28	0.022	4.6	0.088	1.3	2.6	25	1.6
0.23	64									106	6.1	
3.5	272	18	0.27	7.8	20	1.1	37	2.7	24	651	1310	Not Detected
<LOW>	0.59	<LOW>	0.0013	<LOW>	<LOW>	<LOW>	<LOW>	0.30	<LOW>	<LOW>	11	<LOW>
<LOW>	1.1	0.69	0.0026	0.47	<LOW>	<LOW>	<LOW>	0.26	<LOW>	0.99	16	0.55
<LOW>	1.9	0.11	0.0097	1.9	0.21	<LOW>	<LOW>	0.68	0.92	1.5	88	0.27
<LOW>	1.6	<LOW>	0.0070	2.5	<LOW>	0.17	<LOW>	0.67	1.1	<LOW>	88	<LOW>
<LOW>	0.76	1.2	0.0035	0.47	<LOW>	<LOW>	<LOW>	0.30	0.44	2.0	25	4.4
<LOW>	0.42	0.11	0.0013	<LOW>	<LOW>	<LOW>	<LOW>	0.18	0.92	<LOW>	8.3	0.55
<LOW>	1.4	<LOW>	0.0066	1.6	<LOW>	0.037	<LOW>	0.55	<LOW>	0.99	83	0.27

Interleukin-23 (IL-23)	Interleukin-3 (IL-3)	Interleukin-4 (IL-4)	Interleukin-5 (IL-5)	Interleukin-6 (IL-6)	Interleukin-7 (IL-7)	Interleukin-8 (IL-8)	Monocyte Chemotactic Protein 1 (MCP-1)	Macrophage Inflammatory Protein-1 alpha (MIP-1 alpha)	Macrophage Inflammatory Protein-1 beta (MIP-1 beta)	Matrix Metalloproteinase-2 (MMP-2)	Matrix Metalloproteinase-3 (MMP-3)	Matrix Metalloproteinase-9 (MMP-9)
ng/mL	ng/mL	pg/mL	pg/mL	pg/mL	pg/mL	pg/mL	pg/mL	pg/mL	pg/mL	ng/mL	ng/mL	ng/mL
0.16	0.0040	3.6	0.69	0.82	1.5	0.38	0.96	4.6	2.4	1.2	0.010	4.1
							36		15		2.2	
5.9	1.1	32	34	43	60	236	815	61	719	151	51	318
1.1	0.0012	<LOW>	<LOW>	0.66	<LOW>	220	414	1.9	<LOW>	1.8	0.13	5.0
1.8	0.0012	1.1	<LOW>	24	<LOW>	1290	9450	3.2	<LOW>	25	0.25	10
3.5	<LOW>	0.66	<LOW>	167	<LOW>	3100	40	1.9	<LOW>	0.73	0.60	23
3.8	<LOW>	<LOW>	<LOW>	5.8	<LOW>	332	<LOW>	1.9	<LOW>	<LOW>	0.73	27
2.1	<LOW>	6.3	<LOW>	4850	1.3	16500	6.3	10	<LOW>	5.4	0.23	6.2
0.76	<LOW>	<LOW>	<LOW>	0.32	1.3	2.4	630	1.9	<LOW>	3.1	0.14	5.0
3.8	<LOW>	<LOW>	<LOW>	5.8	<LOW>	401	<LOW>	1.9	<LOW>	<LOW>	0.54	20

T-Cell-Specific Protein RANTES (RANTES)	Stem Cell Factor (SCF)	Tissue Inhibitor of Metalloproteinases 1 (TIMP-1)	Tumor Necrosis Factor alpha (TNF-alpha)	Tumor Necrosis Factor beta (TNF-beta)	Tumor Necrosis Factor Receptor 2 (TNFR2)	Vascular Cell Adhesion Molecule-1 (VCAM-1)	Vitamin D-Binding Protein (VDBP)	Vascular Endothelial Growth Factor (VEGF)	von Willebrand Factor (vWF)
ng/mL	pg/mL	ng/mL	pg/mL	pg/mL	ng/mL	ng/mL	ug/mL	pg/mL	ug/mL
0.00030	20	0.019	0.21	12	0.0018	0.0085	3.2E-05	1.8	0.00071
0.94		71			3.6	386	pending	107	1.8
63	276	322	34	28	12	865	pending	1010	42
0.00054	16	22	<LOW>	2.6	<LOW>	0.024	<LOW>	709	0.00082
0.0059	26	23	0.31	7.0	0.0026	0.038	<LOW>	1400	0.00034
0.0037	100	22	0.31	7.0	0.0061	0.031	<LOW>	5680	0.00049
0.00035	110	13	<LOW>	2.6	<LOW>	0.029	<LOW>	8700	0.00034
0.00087	33	81	1.5	79	0.0016	0.036	<LOW>	1560	0.0012
0.038	13	4.4	<LOW>	<LOW>	0.0017	0.015	<LOW>	545	0.00082
0.00016	91	20	<LOW>	<LOW>	0.11	0.032	<LOW>	6120	0.00074
