# Supplementary material for: STAT3 Is Activated by JAK2 Independent of Key Oncogenic Driver Mutations in Non-Small Cell Lung Carcinoma
Source: PLoS One. 2012 Feb 2;7(2):e30820. doi: 10.1371/journal.pone.0030820 (PMC3271110; doi:10.1371/journal.pone.0030820)
Supplement: Table S3 — Patient disease characteristics and outcome statistics. Key clinical, histological and treatment features, as well as outcome statistics, of the patient population represented on our tissue microarray are indicated. Mutational status for KRAS and EGFR are also shown for this population. (PDF) [file pone.0030820.s005.pdf]

Clinical factors		Results
Median Age, yr (Range) N=297		70.4 (34.6-93.4)
Gender (%) N=297		
	Female	161 (54.2)
	Male	136 (45.8)
Disease stage (%) N=297		
	IA	131 (44.1)
	IB	96 (32.3)
	2A	47 (15.8)
	2B	23 (7.8)
Histology (%) N=297		
	Adenocarcinoma	187 (63.0)
	Squamous cell carcinoma	84 (28.3)
	Large cell carcinoma	10 (3.4)
	BAC*	9 (3.0)
	NSCLC-NOS**	7 (2.3)
Smoking status (%) N=283		
	Smokers	262 (92.6)
	Never smokers	21 (7.4)
Median pack/year (Range) N=141		50 (6-200)
How Chemotherapy Delivered (%) N=42		
	Neoadjuvant	1 (2.4)
	Adjuvant	35 (83.3)
	Concurrent with radiation	6 (14.3)
Type of Chemotherapy Delivered (%) N=42		
	Carboplatin-based regimen	13 (31.0)
	Cisplatin-based regimen	5 (11.9)
	Other	24 (57.1)
Radiation therapy (%) N=38		
	Definitive radiation	29 (76.3)
	Adjuvant radiation	9 (23.7)
**Outcome & Mutational Statistics**		
Median Overall Survival, in months (range) N=296		38.8 (0.4-110.5)
Disease recurrence? N=270		
	No	145 (53.7)
	Yes	125 (46.3)
Median Relapse-free Survival, in months (range) N=270		31.7 (0.4-110.5)
Immunohistochemistry (% positive)		
	STAT3 N = 245	229 (93.5)
	p-STAT3^{Y705} N=245	53 (21.6)
	Jak2 N= 245	194 (79.2)
Mutational status (%) N = 270		
	K-Ras mutation, exon 1 codons 12 and 13	70 (26.0)
	EGFR mutation, exon 19	15 (5.6)

*BAC: bronchoalveolar carcinoma
**NOS: non-otherwise specified

Supplemental Table S3. Patient disease characteristics and outcome statistics. Key clinical, histological and treatment features, as well as outcome statistics, of the patient population represented on our tissue microarray are indicated. Mutational status for KRAS and EGFR are also shown for this population.
