# Supplementary material for: STAT3 Is Activated by JAK2 Independent of Key Oncogenic Driver Mutations in Non-Small Cell Lung Carcinoma
Source: PLoS One. 2012 Feb 2;7(2):e30820. doi: 10.1371/journal.pone.0030820 (PMC3271110; doi:10.1371/journal.pone.0030820)
Supplement: Table S4 — Primers for IL-6 Family q-RT-PCR. Oligonucleotide sequences of each primer pair (forward and reverse, 5′-to-3′) used in quantitative RT-PCR detection of IL-6 family ligands are shown. Each pair was validated to produce a single PCR fragment of the expected length by gel electrophoresis. (PDF) [file pone.0030820.s006.pdf]

	Forward	Reverse
**IL-6**	AAATTCGGTACATCCTCGACGGCA	AGTGCCTCTTTGCTGCTTTCACAC
**IL-11**	ACAGCTGAGGGACAAATTCCCA	TCAGCACACCTGGGAGCTGTAGA
**LIF**	CATGAACCAGATCAGGAGCCAACT	GCCACATAGCTTGTCCAGGTTGTT
**OSM**	AGCTCCAGAAGCAGACAGATCTCA	TCCCTGCAGTGCTCTCTCAGTTTA
**CTF1**	AAGATCCGTCAGACACACAGCCTT	TCCCTGGAGCTGCACATATTCCT
**CNTF**	ATGGCTTTCACAGAGCATTCACCG	ATCCGCAGAGTCCAGGTTGATGTT
**CLCF1**	AAACCTATGACCTCACCCGCTACCT	AGGGTTGAAGTCTGGCTCGTTGAA
**CRLF1**	AACATCAGCTGCTGGTCCAAGAAC	TGTTGTCCTGGCCATACCACCTAA

Supplemental Table S4. Primers for IL-6 Family q-RT-PCR
Oligonucleotide sequences of each primer pair (forward and reverse, 5’-to-3’) used in quantitative RT-PCR detection of IL-6 family ligands are shown. Each pair was validated to produce a single PCR fragment of the expected length by gel electrophoresis.
